# 3-(Adamantan-1-yl)-1-[(4-benzyl­piperazin-1-yl)meth­yl]-4-[(*E*)-(2-hy­droxy­benzyl­idene)amino]-1*H*-1,2,4-triazole-5(4*H*)-thione

**DOI:** 10.1107/S1600536812021204

**Published:** 2012-05-19

**Authors:** Ali A. El-Emam, Mohamed A. Al-Omar, Abdul-Malek S. Al-Tamimi, Seik Weng Ng, Edward R. T. Tiekink

**Affiliations:** a Department of Pharmaceutical Chemistry, College of Pharmacy, King Saud University, Riyadh 11451, Saudi Arabia; b Department of Chemistry, University of Malaya, 50603 Kuala Lumpur, Malaysia; c Chemistry Department, Faculty of Science, King Abdulaziz University, PO Box 80203 Jeddah, Saudi Arabia

## Abstract

In the title compound, C_31_H_38_N_6_OS, the conformation about the N=C [1.285 (2) Å] imine bond is *E*. The piperazine ring has a chair conformation and occupies a position almost perpendicular to the plane through the triazole ring; the benzene ring forms a dihedral angle of 31.95 (10)° with the triazole ring. Overall, the mol­ecule has the shape of a flattened bowl. The hy­droxy group is disordered over two positions. The major component has a site-occupancy factor of 0.762 (3) and forms an intra­molecular O—H⋯N(imine) bond to close an *S*(6) loop. The minor component of the disordered hy­droxy group forms an O—H⋯N(piperazine) hydrogen bond. These, along with C—H⋯S and C—H⋯N inter­actions, link mol­ecules into a three-dimensional architecture.

## Related literature

For the diverse biological activities of adamantane derivatives, see: Vernier *et al.* (1969 ▶); El-Emam *et al.* (2004 ▶); Kadi *et al.* (2007 ▶, 2010 ▶). For related structural studies, see: Kadi *et al.* (2011 ▶); El-Emam *et al.* (2012 ▶). For the synthesis of the precursor to the title compound, see: Al-Omar *et al.* (2010 ▶)
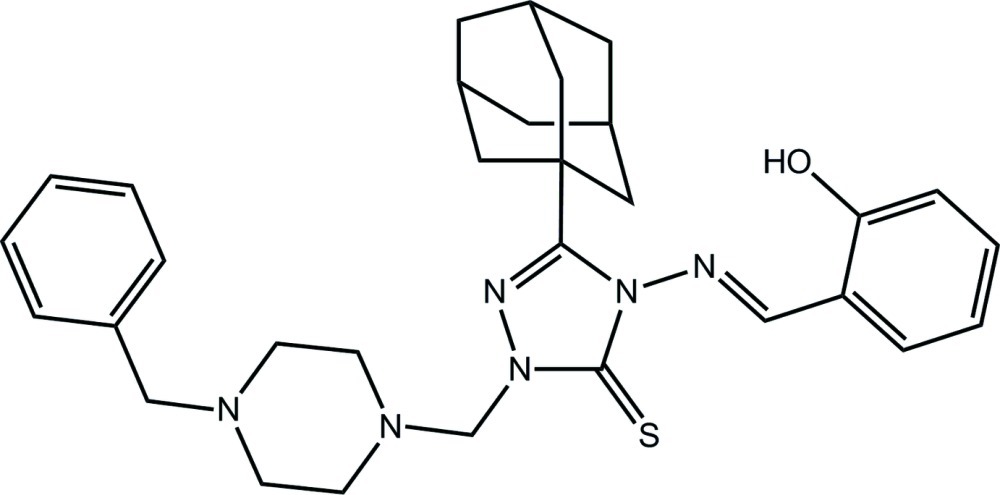


## Experimental

### 

#### Crystal data

C_31_H_38_N_6_OS
*M* *_r_* = 542.73
Monoclinic, 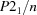

*a* = 10.6015 (5) Å
*b* = 12.0283 (7) Å
*c* = 22.7865 (12) Å
β = 101.222 (4)°
*V* = 2850.1 (3) Å^3^
*Z* = 4
Mo *K*α radiation
μ = 0.15 mm^−1^
*T* = 100 K
0.40 × 0.40 × 0.10 mm

#### Data collection

Agilent SuperNova Dual diffractometer with an Atlas detector
Absorption correction: multi-scan (*CrysAlis PRO*; Agilent, 2011 ▶) *T*_min_ = 0.570, *T*_max_ = 1.000
11447 measured reflections
6530 independent reflections
4526 reflections with *I* > 2σ(*I*)
*R*_int_ = 0.032

#### Refinement

*R*[*F* ^2^ > 2σ(*F* ^2^)] = 0.052
*wR*(*F* ^2^) = 0.125
*S* = 1.02
6530 reflections
364 parameters
H-atom parameters constrained
Δρ_max_ = 0.28 e Å^−3^
Δρ_min_ = −0.29 e Å^−3^

### 

Data collection: *CrysAlis PRO* (Agilent, 2011 ▶); cell refinement: *CrysAlis PRO*; data reduction: *CrysAlis PRO*; program(s) used to solve structure: *SHELXS97* (Sheldrick, 2008 ▶); program(s) used to refine structure: *SHELXL97* (Sheldrick, 2008 ▶); molecular graphics: *ORTEP-3* (Farrugia, 1997 ▶) and *DIAMOND* (Brandenburg, 2006 ▶); software used to prepare material for publication: *publCIF* (Westrip, 2010 ▶).

## Supplementary Material

Crystal structure: contains datablock(s) global, I. DOI: 10.1107/S1600536812021204/qm2068sup1.cif

Structure factors: contains datablock(s) I. DOI: 10.1107/S1600536812021204/qm2068Isup2.hkl

Supplementary material file. DOI: 10.1107/S1600536812021204/qm2068Isup3.cml

Additional supplementary materials:  crystallographic information; 3D view; checkCIF report

## Figures and Tables

**Table 1:** Hydrogen-bond geometry (Å, °)

*D*—H⋯*A*	*D*—H	H⋯*A*	*D*⋯*A*	*D*—H⋯*A*
O1—H1*o*⋯N1	0.84	1.89	2.632 (2)	147
O1′—H1*o*′⋯N5^i^	0.84	1.92	2.714 (6)	158
C13—H13*A*⋯S1^ii^	0.99	2.68	3.650 (2)	166
C30—H30⋯N6^iii^	0.95	2.54	3.484 (3)	172

Symmetry codes: (i) 
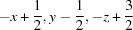
; (ii) 
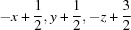
; (iii) 
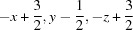
.

**Fig. 1.**
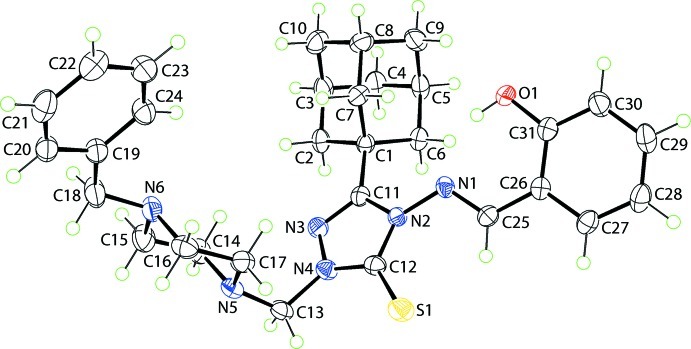
The molecular structure of (I) showing the atom-labelling scheme and displacement ellipsoids at the 50% probability level.

**Fig. 2.**
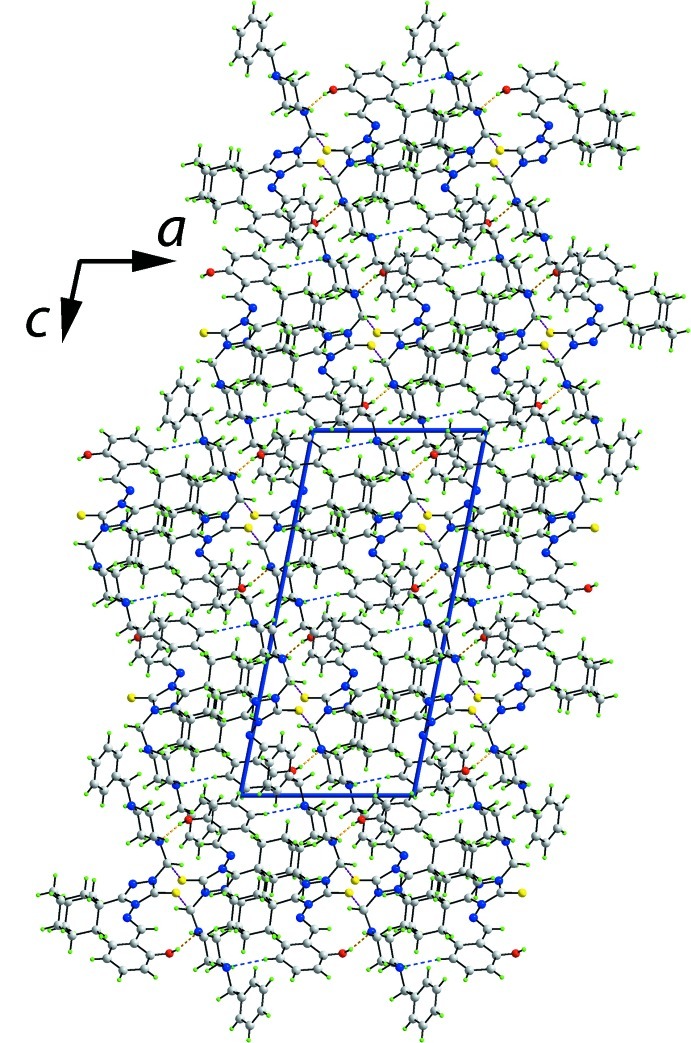
A view in projection down the b axis of the unit-cell contents for (I). The O—H···S, C—H···S and C—H···N are shown as blue, orange, and purple dashed lines, respectively.

## supplementary crystallographic information

### Comment

Derivatives of adamantane have long been known for their diverse biological activities including anti-viral activity against the influenza (Vernier *et al.*, 1969) and HIV viruses (El-Emam *et al.*, 2004). Moreover, adamantane derivatives were reported to exhibit marked anti-bacterial and anti-inflammatory activities (Kadi *et al.*, 2007; Kadi *et al.*, 2010). In continuation of our interest in the chemical and pharmacological properties of adamantane derivatives, and as part of on-going structural studies (Kadi *et al.*, 2011; El-Emam *et al.*, 2012), we synthesized the title compound (I) as a potential chemotherapeutic agent. Herein, we describe the crystal and molecular structure of (I).

In (I), Fig. 1, the conformation about the N1═C25 [1.285 (2) Å] imine bond is *E*. The piperazinyl ring, having a chair conformation, projects almost normal to the plane through the triazole ring (r.m.s. deviation = 0.014 Å) as seen in the value of the N3—N4—C13—N5 torsion angle = -67.3 (2)°. By contrast, the benzene ring is splayed with respect to the triazole ring with the C25—N1—N2—C11 torsion angle being -153.92 (17)°; the dihedral angle between the rings is 31.95 (10)°. Overall, the molecule has the shape of a flattened bowl. As noted below, the hydroxy group is disordered over two positions. The major component is aligned to allow the formation of an intramolecular *O*—*H*···*N*(imine) bond to close an *S*(6) loop, Table 1.

In the crystal packing, the minor component of the disordered hydroxy group forms an O—H···N(piperazinyl) hydrogen bond; Table 1. Additional links between molecules are of the type C—H···S and C—H···N, Table 1, to consolidate the crystal packing, Fig. 2.

### Experimental

A mixture of 3-(1-adamantyl)-4-(2-hydroxybenzylideneamino)-4*H*-1,2,4-triazole-5-thiol (709 mg, 2 mmol), prepared following literature methods (Al-Omar *et al.*, 2010), 1-benzylpiperazine (353 mg, 2 mmol) and 37% formaldehyde solution (1 ml), in ethanol (8 ml), was heated under reflux for 15 min. when a clear solution was obtained. Stirring was continued for 12 h. at room temperature and the mixture was allowed to stand overnight. Cold water (5 ml) was added and the mixture was stirred for 20 min. The precipitated crude product was filtered, washed with water, dried, and crystallized from ethanol to yield 413 mg (38%) of the title compound as colourless crystals. *M*.pt: 437–439 K. Crystals were obtained by slow evaporation of its CHCl_3_:EtOH (1:1; 5 ml) solution at room temperature. ^1^H NMR (DMSO-d_6_, 500.13 MHz): δ 1.71 (br. s, 6H, adamantane-H), 2.04 (s, 3H, adamantane-H), 2.09 (s, 6H, adamantane-H), 2.37 (br. s, 4H, piperazine-H), 2.72 (s, 4H, piperazine-H), 3.49 (s, 2H, PhCH_2_), 5.16 (s, 2H, CH_2_), 6.98–7.03 (m, 2H, Ar—H), 7.23–7.32 (m, 5H, Ar—H), 7.44–7.47 (m, 2H, Ar—H), 9.85 (s, 1H, CH═ N), 10.50 (br. s, 1H, OH) p.p.m. ^13^C NMR (DMSO-d_6_, 125.76 MHz): δ 27.18, 34.73, 35.92, 38.10 (adamantane-C), 49.93, 52.47 (piperazine-C), 62.08 (PhCH_2_), 68.69 (CH_2_), 116.77, 118.28, 119.80, 126.68, 126.85, 128.09, 128.82, 134.44, 138.01, 158.56 (Ar—C), 154.20 (triazole C-5), 162.01 p.p.m.

### Refinement

The H-atoms were placed in calculated positions [O—H = 0.84 Å and C—H = 0.95 to 1.00 Å, *U*_iso_(H) = 1.2–1.5*U*_eq_(O,*C*)] and were included in the refinement in the riding model approximation. The hydroxy group was disordered over two positions. Each component was refined independently and the major component has a site occupancy factor = 0.762 (3). A reflection, *i.e*. (12 2 8), was omitted from the final cycles of refinement owing to poor agreement.

### Crystal data

**Table:**

C_31_H_38_N_6_OS	*F*(000) = 1160
*M**_r_* = 542.73	*D*_x_ = 1.265 Mg m^−^^3^
Monoclinic, *P*2_1_/*n*	Mo *K*α radiation, λ = 0.71073 Å
Hall symbol: -P 2yn	Cell parameters from 3530 reflections
*a* = 10.6015 (5) Å	θ = 2.3–27.5°
*b* = 12.0283 (7) Å	µ = 0.15 mm^−^^1^
*c* = 22.7865 (12) Å	*T* = 100 K
β = 101.222 (4)°	Prism, colourless
*V* = 2850.1 (3) Å^3^	0.40 × 0.40 × 0.10 mm
*Z* = 4	

### Data collection

**Table:**

Agilent SuperNova Dual diffractometer with an Atlas detector	6530 independent reflections
Radiation source: SuperNova (Mo) X-ray Source	4526 reflections with *I* > 2σ(*I*)
Mirror monochromator	*R*_int_ = 0.032
Detector resolution: 10.4041 pixels mm^-1^	θ_max_ = 27.6°, θ_min_ = 2.3°
ω scan	*h* = −13→9
Absorption correction: multi-scan (*CrysAlis PRO*; Agilent, 2011)	*k* = −10→15
*T*_min_ = 0.570, *T*_max_ = 1.000	*l* = −20→29
11447 measured reflections	

### Refinement

**Table:**

Refinement on *F*^2^	Primary atom site location: structure-invariant direct methods
Least-squares matrix: full	Secondary atom site location: difference Fourier map
*R*[*F*^2^ > 2σ(*F*^2^)] = 0.052	Hydrogen site location: inferred from neighbouring sites
*wR*(*F*^2^) = 0.125	H-atom parameters constrained
*S* = 1.02	*w* = 1/[σ^2^(*F*_o_^2^) + (0.042*P*)^2^ + 0.843*P*] where *P* = (*F*_o_^2^ + 2*F*_c_^2^)/3
6530 reflections	(Δ/σ)_max_ = 0.001
364 parameters	Δρ_max_ = 0.28 e Å^−^^3^
0 restraints	Δρ_min_ = −0.29 e Å^−^^3^

### Special details

**Table:**

Refinement. Refinement of *F*^2^ against ALL reflections. The weighted *R*-factor *wR* and goodness of fit *S* are based on *F*^2^, conventional *R*-factors *R* are based on *F*, with *F* set to zero for negative *F*^2^. The threshold expression of *F*^2^ > σ(*F*^2^) is used only for calculating *R*-factors(gt) *etc*. and is not relevant to the choice of reflections for refinement. *R*-factors based on *F*^2^ are statistically about twice as large as those based on *F*, and *R*- factors based on ALL data will be even larger.

### Fractional atomic coordinates and isotropic or equivalent isotropic displacement parameters (Å^2^)

**Table:**

	*x*	*y*	*z*	*U*_iso_*/*U*_eq_	Occ. (<1)
S1	0.26003 (5)	0.62548 (5)	0.73271 (3)	0.03659 (16)	
O1	0.67165 (14)	0.49724 (17)	0.61888 (8)	0.0329 (6)	0.762 (3)
H1o	0.6498	0.5466	0.6410	0.049*	0.762 (3)
O1'	0.2282 (5)	0.4574 (5)	0.5670 (3)	0.0324 (18)	0.238 (3)
H1o'	0.1859	0.4049	0.5780	0.049*	0.238 (3)
N1	0.51345 (13)	0.62441 (13)	0.66474 (7)	0.0240 (4)	
N2	0.48195 (13)	0.70959 (13)	0.70132 (7)	0.0222 (3)	
N3	0.51922 (14)	0.86006 (14)	0.75723 (7)	0.0261 (4)	
N4	0.40900 (13)	0.80618 (14)	0.76589 (7)	0.0265 (4)	
N5	0.41461 (14)	0.82682 (15)	0.87387 (7)	0.0280 (4)	
N6	0.61744 (15)	0.77152 (15)	0.97143 (7)	0.0307 (4)	
C1	0.68951 (16)	0.82020 (17)	0.69971 (8)	0.0229 (4)	
C2	0.74105 (17)	0.93454 (18)	0.72398 (9)	0.0290 (4)	
H2A	0.6806	0.9936	0.7061	0.035*	
H2B	0.7479	0.9366	0.7679	0.035*	
C3	0.87413 (18)	0.95560 (19)	0.70855 (9)	0.0351 (5)	
H3	0.9070	1.0298	0.7245	0.042*	
C4	0.8622 (2)	0.9539 (2)	0.64047 (9)	0.0386 (5)	
H4A	0.8029	1.0133	0.6221	0.046*	
H4B	0.9474	0.9677	0.6302	0.046*	
C5	0.81110 (19)	0.84063 (19)	0.61607 (9)	0.0348 (5)	
H5	0.8038	0.8395	0.5716	0.042*	
C6	0.67807 (17)	0.82016 (18)	0.63125 (8)	0.0278 (4)	
H6A	0.6439	0.7477	0.6146	0.033*	
H6B	0.6177	0.8792	0.6132	0.033*	
C7	0.78629 (16)	0.73016 (18)	0.72866 (9)	0.0281 (4)	
H7A	0.7946	0.7321	0.7727	0.034*	
H7B	0.7543	0.6557	0.7144	0.034*	
C8	0.91771 (17)	0.7508 (2)	0.71237 (10)	0.0364 (5)	
H8	0.9793	0.6915	0.7304	0.044*	
C9	0.90309 (18)	0.7490 (2)	0.64414 (10)	0.0380 (5)	
H9A	0.9881	0.7602	0.6332	0.046*	
H9B	0.8695	0.6758	0.6285	0.046*	
C10	0.96774 (19)	0.8648 (2)	0.73648 (10)	0.0388 (6)	
H10A	0.9768	0.8661	0.7805	0.047*	
H10B	1.0534	0.8785	0.7267	0.047*	
C11	0.56314 (16)	0.79885 (16)	0.71860 (8)	0.0231 (4)	
C12	0.38254 (16)	0.71301 (17)	0.73315 (8)	0.0261 (4)	
C13	0.34350 (17)	0.84426 (19)	0.81387 (9)	0.0301 (5)	
H13A	0.3250	0.9247	0.8083	0.036*	
H13B	0.2601	0.8051	0.8095	0.036*	
C14	0.52571 (18)	0.90045 (18)	0.89159 (9)	0.0335 (5)	
H14A	0.4996	0.9786	0.8827	0.040*	
H14B	0.5932	0.8815	0.8687	0.040*	
C15	0.5775 (2)	0.88674 (19)	0.95780 (10)	0.0386 (5)	
H15A	0.6520	0.9369	0.9703	0.046*	
H15B	0.5103	0.9075	0.9805	0.046*	
C16	0.50592 (17)	0.6981 (2)	0.95405 (9)	0.0339 (5)	
H16A	0.4395	0.7170	0.9775	0.041*	
H16B	0.5323	0.6199	0.9628	0.041*	
C17	0.45078 (17)	0.71111 (18)	0.88788 (9)	0.0298 (5)	
H17A	0.5154	0.6875	0.8644	0.036*	
H17B	0.3742	0.6628	0.8766	0.036*	
C18	0.6755 (2)	0.7621 (2)	1.03506 (9)	0.0417 (6)	
H18A	0.6078	0.7740	1.0588	0.050*	
H18B	0.7399	0.8220	1.0455	0.050*	
C19	0.74004 (17)	0.65195 (19)	1.05285 (9)	0.0310 (5)	
C20	0.75362 (17)	0.6155 (2)	1.11188 (9)	0.0344 (5)	
H20	0.7175	0.6578	1.1397	0.041*	
C21	0.81892 (19)	0.5186 (2)	1.13025 (9)	0.0405 (6)	
H21	0.8286	0.4956	1.1708	0.049*	
C22	0.87039 (19)	0.4547 (2)	1.09039 (10)	0.0410 (6)	
H22	0.9145	0.3875	1.1031	0.049*	
C23	0.85679 (18)	0.4899 (2)	1.03140 (10)	0.0382 (5)	
H23	0.8914	0.4465	1.0035	0.046*	
C24	0.79293 (18)	0.5884 (2)	1.01311 (9)	0.0347 (5)	
H24	0.7854	0.6125	0.9729	0.042*	
C25	0.41941 (16)	0.57102 (17)	0.63287 (8)	0.0248 (4)	
H25	0.3335	0.5914	0.6343	0.030*	
C26	0.44301 (16)	0.48029 (17)	0.59477 (8)	0.0240 (4)	
C27	0.33739 (17)	0.42259 (18)	0.56174 (8)	0.0283 (5)	
H27	0.2529	0.4435	0.5654	0.034*	0.762 (3)
C28	0.35374 (19)	0.3367 (2)	0.52428 (9)	0.0359 (5)	
H28	0.2811	0.3001	0.5013	0.043*	
C29	0.47679 (19)	0.3037 (2)	0.52021 (9)	0.0370 (5)	
H29	0.4883	0.2441	0.4944	0.044*	
C30	0.58288 (19)	0.35642 (19)	0.55309 (9)	0.0341 (5)	
H30	0.6669	0.3317	0.5508	0.041*	
C31	0.56677 (17)	0.44516 (18)	0.58934 (8)	0.0276 (4)	
H31	0.6401	0.4829	0.6109	0.033*	0.238 (3)

### Atomic displacement parameters (Å^2^)

**Table:**

	*U*^11^	*U*^22^	*U*^33^	*U*^12^	*U*^13^	*U*^23^
S1	0.0313 (3)	0.0351 (3)	0.0467 (3)	−0.0086 (2)	0.0159 (2)	−0.0070 (3)
O1	0.0223 (9)	0.0354 (13)	0.0382 (11)	−0.0003 (8)	−0.0007 (7)	−0.0151 (10)
O1'	0.022 (3)	0.035 (4)	0.039 (4)	−0.011 (3)	0.001 (2)	0.004 (3)
N1	0.0281 (8)	0.0193 (9)	0.0243 (8)	0.0006 (7)	0.0045 (6)	−0.0031 (7)
N2	0.0220 (7)	0.0188 (9)	0.0253 (8)	0.0006 (6)	0.0035 (6)	−0.0023 (7)
N3	0.0268 (8)	0.0241 (10)	0.0272 (8)	0.0007 (7)	0.0049 (6)	−0.0011 (8)
N4	0.0265 (8)	0.0235 (10)	0.0303 (9)	0.0012 (7)	0.0077 (6)	−0.0013 (8)
N5	0.0276 (8)	0.0261 (10)	0.0319 (9)	0.0049 (7)	0.0098 (6)	−0.0022 (8)
N6	0.0379 (9)	0.0291 (11)	0.0245 (8)	0.0028 (8)	0.0045 (6)	−0.0043 (8)
C1	0.0238 (9)	0.0221 (11)	0.0226 (9)	−0.0035 (8)	0.0043 (7)	−0.0013 (8)
C2	0.0367 (10)	0.0246 (11)	0.0257 (10)	−0.0061 (9)	0.0060 (8)	−0.0048 (9)
C3	0.0399 (11)	0.0299 (13)	0.0362 (12)	−0.0135 (9)	0.0090 (8)	−0.0107 (10)
C4	0.0456 (12)	0.0371 (14)	0.0355 (12)	−0.0161 (10)	0.0141 (9)	−0.0040 (11)
C5	0.0422 (11)	0.0376 (14)	0.0274 (11)	−0.0156 (10)	0.0133 (8)	−0.0103 (10)
C6	0.0323 (10)	0.0268 (12)	0.0236 (10)	−0.0056 (8)	0.0038 (7)	−0.0013 (9)
C7	0.0250 (9)	0.0281 (12)	0.0294 (10)	−0.0006 (8)	0.0006 (7)	0.0009 (9)
C8	0.0239 (10)	0.0380 (14)	0.0457 (13)	−0.0016 (9)	0.0028 (8)	−0.0058 (11)
C9	0.0290 (10)	0.0391 (14)	0.0495 (13)	−0.0104 (9)	0.0166 (9)	−0.0165 (12)
C10	0.0299 (10)	0.0471 (16)	0.0383 (12)	−0.0105 (10)	0.0041 (8)	−0.0063 (12)
C11	0.0257 (9)	0.0207 (11)	0.0214 (9)	0.0021 (8)	0.0008 (7)	0.0022 (8)
C12	0.0242 (9)	0.0266 (12)	0.0274 (10)	0.0033 (8)	0.0052 (7)	0.0021 (9)
C13	0.0282 (10)	0.0295 (12)	0.0340 (11)	0.0085 (9)	0.0094 (8)	−0.0026 (10)
C14	0.0407 (11)	0.0219 (12)	0.0376 (12)	0.0025 (9)	0.0068 (8)	−0.0055 (10)
C15	0.0472 (12)	0.0302 (13)	0.0372 (12)	0.0036 (10)	0.0055 (9)	−0.0079 (11)
C16	0.0319 (10)	0.0371 (14)	0.0354 (11)	0.0039 (9)	0.0134 (8)	0.0050 (11)
C17	0.0265 (9)	0.0278 (12)	0.0356 (11)	−0.0005 (8)	0.0074 (8)	−0.0021 (10)
C18	0.0562 (13)	0.0440 (16)	0.0245 (11)	0.0063 (11)	0.0072 (9)	−0.0062 (11)
C19	0.0303 (10)	0.0360 (13)	0.0270 (10)	−0.0021 (9)	0.0061 (7)	−0.0032 (10)
C20	0.0299 (10)	0.0482 (15)	0.0255 (10)	−0.0077 (10)	0.0064 (8)	−0.0057 (11)
C21	0.0436 (12)	0.0486 (16)	0.0270 (11)	−0.0115 (11)	0.0015 (9)	0.0031 (11)
C22	0.0397 (12)	0.0388 (15)	0.0405 (13)	−0.0021 (10)	−0.0019 (9)	0.0027 (12)
C23	0.0368 (11)	0.0407 (15)	0.0376 (12)	−0.0005 (10)	0.0088 (9)	−0.0040 (11)
C24	0.0377 (11)	0.0416 (14)	0.0262 (10)	−0.0008 (10)	0.0097 (8)	−0.0006 (10)
C25	0.0248 (9)	0.0248 (11)	0.0235 (9)	−0.0002 (8)	0.0020 (7)	0.0033 (9)
C26	0.0292 (9)	0.0205 (11)	0.0217 (9)	−0.0020 (8)	0.0037 (7)	0.0014 (8)
C27	0.0283 (10)	0.0304 (12)	0.0254 (10)	−0.0076 (8)	0.0035 (7)	0.0021 (9)
C28	0.0397 (11)	0.0362 (13)	0.0307 (11)	−0.0144 (10)	0.0041 (8)	−0.0065 (11)
C29	0.0477 (12)	0.0324 (13)	0.0322 (11)	−0.0070 (10)	0.0107 (9)	−0.0106 (11)
C30	0.0372 (11)	0.0314 (13)	0.0344 (11)	−0.0005 (9)	0.0090 (8)	−0.0060 (10)
C31	0.0300 (10)	0.0258 (11)	0.0259 (10)	−0.0032 (8)	0.0026 (7)	−0.0008 (9)

### Geometric parameters (Å, º)

**Table:**

S1—C12	1.670 (2)	C9—H9A	0.9900
O1—C31	1.338 (2)	C9—H9B	0.9900
O1—H1o	0.8400	C10—H10A	0.9900
O1'—C27	1.259 (6)	C10—H10B	0.9900
O1'—H1o'	0.8400	C13—H13A	0.9900
N1—C25	1.285 (2)	C13—H13B	0.9900
N1—N2	1.402 (2)	C14—C15	1.511 (3)
N2—C11	1.384 (2)	C14—H14A	0.9900
N2—C12	1.391 (2)	C14—H14B	0.9900
N3—C11	1.301 (2)	C15—H15A	0.9900
N3—N4	1.384 (2)	C15—H15B	0.9900
N4—C12	1.345 (3)	C16—C17	1.515 (3)
N4—C13	1.477 (2)	C16—H16A	0.9900
N5—C13	1.442 (2)	C16—H16B	0.9900
N5—C17	1.462 (3)	C17—H17A	0.9900
N5—C14	1.466 (3)	C17—H17B	0.9900
N6—C18	1.465 (3)	C18—C19	1.509 (3)
N6—C15	1.465 (3)	C18—H18A	0.9900
N6—C16	1.467 (3)	C18—H18B	0.9900
C1—C11	1.507 (2)	C19—C24	1.384 (3)
C1—C6	1.541 (3)	C19—C20	1.395 (3)
C1—C2	1.542 (3)	C20—C21	1.378 (3)
C1—C7	1.548 (3)	C20—H20	0.9500
C2—C3	1.540 (3)	C21—C22	1.381 (3)
C2—H2A	0.9900	C21—H21	0.9500
C2—H2B	0.9900	C22—C23	1.390 (3)
C3—C10	1.529 (3)	C22—H22	0.9500
C3—C4	1.532 (3)	C23—C24	1.387 (3)
C3—H3	1.0000	C23—H23	0.9500
C4—C5	1.530 (3)	C24—H24	0.9500
C4—H4A	0.9900	C25—C26	1.446 (3)
C4—H4B	0.9900	C25—H25	0.9500
C5—C9	1.527 (3)	C26—C27	1.405 (3)
C5—C6	1.536 (2)	C26—C31	1.407 (2)
C5—H5	1.0000	C27—C28	1.373 (3)
C6—H6A	0.9900	C27—H27	0.9500
C6—H6B	0.9900	C28—C29	1.384 (3)
C7—C8	1.530 (3)	C28—H28	0.9500
C7—H7A	0.9900	C29—C30	1.378 (3)
C7—H7B	0.9900	C29—H29	0.9500
C8—C10	1.533 (3)	C30—C31	1.381 (3)
C8—C9	1.532 (3)	C30—H30	0.9500
C8—H8	1.0000	C31—H31	0.9500
			
C31—O1—H1o	109.5	N2—C12—S1	130.17 (15)
C27—O1'—H1o'	109.5	N5—C13—N4	114.97 (15)
C25—N1—N2	116.93 (15)	N5—C13—H13A	108.5
C11—N2—C12	108.71 (15)	N4—C13—H13A	108.5
C11—N2—N1	121.94 (14)	N5—C13—H13B	108.5
C12—N2—N1	128.61 (15)	N4—C13—H13B	108.5
C11—N3—N4	104.76 (15)	H13A—C13—H13B	107.5
C12—N4—N3	113.70 (14)	N5—C14—C15	109.01 (18)
C12—N4—C13	126.25 (16)	N5—C14—H14A	109.9
N3—N4—C13	119.47 (16)	C15—C14—H14A	109.9
C13—N5—C17	114.32 (17)	N5—C14—H14B	109.9
C13—N5—C14	114.64 (17)	C15—C14—H14B	109.9
C17—N5—C14	110.63 (15)	H14A—C14—H14B	108.3
C18—N6—C15	109.38 (17)	N6—C15—C14	110.42 (18)
C18—N6—C16	112.46 (17)	N6—C15—H15A	109.6
C15—N6—C16	109.15 (16)	C14—C15—H15A	109.6
C11—C1—C6	112.95 (14)	N6—C15—H15B	109.6
C11—C1—C2	108.80 (15)	C14—C15—H15B	109.6
C6—C1—C2	108.42 (16)	H15A—C15—H15B	108.1
C11—C1—C7	108.15 (15)	N6—C16—C17	109.94 (17)
C6—C1—C7	110.25 (16)	N6—C16—H16A	109.7
C2—C1—C7	108.17 (15)	C17—C16—H16A	109.7
C3—C2—C1	109.95 (16)	N6—C16—H16B	109.7
C3—C2—H2A	109.7	C17—C16—H16B	109.7
C1—C2—H2A	109.7	H16A—C16—H16B	108.2
C3—C2—H2B	109.7	N5—C17—C16	110.46 (18)
C1—C2—H2B	109.7	N5—C17—H17A	109.6
H2A—C2—H2B	108.2	C16—C17—H17A	109.6
C10—C3—C4	109.31 (18)	N5—C17—H17B	109.6
C10—C3—C2	109.67 (18)	C16—C17—H17B	109.6
C4—C3—C2	109.30 (16)	H17A—C17—H17B	108.1
C10—C3—H3	109.5	N6—C18—C19	114.52 (18)
C4—C3—H3	109.5	N6—C18—H18A	108.6
C2—C3—H3	109.5	C19—C18—H18A	108.6
C5—C4—C3	109.49 (18)	N6—C18—H18B	108.6
C5—C4—H4A	109.8	C19—C18—H18B	108.6
C3—C4—H4A	109.8	H18A—C18—H18B	107.6
C5—C4—H4B	109.8	C24—C19—C20	118.6 (2)
C3—C4—H4B	109.8	C24—C19—C18	121.94 (19)
H4A—C4—H4B	108.2	C20—C19—C18	119.41 (19)
C9—C5—C4	109.74 (17)	C21—C20—C19	120.7 (2)
C9—C5—C6	109.24 (17)	C21—C20—H20	119.7
C4—C5—C6	109.68 (17)	C19—C20—H20	119.7
C9—C5—H5	109.4	C20—C21—C22	120.7 (2)
C4—C5—H5	109.4	C20—C21—H21	119.6
C6—C5—H5	109.4	C22—C21—H21	119.6
C5—C6—C1	109.46 (15)	C21—C22—C23	119.1 (2)
C5—C6—H6A	109.8	C21—C22—H22	120.5
C1—C6—H6A	109.8	C23—C22—H22	120.5
C5—C6—H6B	109.8	C24—C23—C22	120.2 (2)
C1—C6—H6B	109.8	C24—C23—H23	119.9
H6A—C6—H6B	108.2	C22—C23—H23	119.9
C8—C7—C1	110.02 (17)	C19—C24—C23	120.7 (2)
C8—C7—H7A	109.7	C19—C24—H24	119.6
C1—C7—H7A	109.7	C23—C24—H24	119.6
C8—C7—H7B	109.7	N1—C25—C26	120.69 (16)
C1—C7—H7B	109.7	N1—C25—H25	119.7
H7A—C7—H7B	108.2	C26—C25—H25	119.7
C7—C8—C10	109.03 (17)	C27—C26—C31	117.66 (18)
C7—C8—C9	109.07 (15)	C27—C26—C25	118.77 (16)
C10—C8—C9	109.61 (19)	C31—C26—C25	123.56 (16)
C7—C8—H8	109.7	O1'—C27—C28	122.6 (3)
C10—C8—H8	109.7	O1'—C27—C26	115.9 (3)
C9—C8—H8	109.7	C28—C27—C26	121.40 (18)
C5—C9—C8	110.21 (18)	C28—C27—H27	119.3
C5—C9—H9A	109.6	C26—C27—H27	119.3
C8—C9—H9A	109.6	C27—C28—C29	119.47 (18)
C5—C9—H9B	109.6	C27—C28—H28	120.3
C8—C9—H9B	109.6	C29—C28—H28	120.3
H9A—C9—H9B	108.1	C30—C29—C28	120.8 (2)
C3—C10—C8	109.80 (16)	C30—C29—H29	119.6
C3—C10—H10A	109.7	C28—C29—H29	119.6
C8—C10—H10A	109.7	C31—C30—C29	119.85 (19)
C3—C10—H10B	109.7	C31—C30—H30	120.1
C8—C10—H10B	109.7	C29—C30—H30	120.1
H10A—C10—H10B	108.2	O1—C31—C30	118.39 (18)
N3—C11—N2	110.28 (16)	O1—C31—C26	120.87 (19)
N3—C11—C1	123.49 (17)	C30—C31—C26	120.74 (17)
N2—C11—C1	126.09 (17)	C30—C31—H31	119.6
N4—C12—N2	102.50 (15)	C26—C31—H31	119.6
N4—C12—S1	127.33 (14)		
			
C25—N1—N2—C11	−153.92 (17)	C11—N2—C12—N4	1.88 (19)
C25—N1—N2—C12	37.0 (3)	N1—N2—C12—N4	172.08 (16)
C11—N3—N4—C12	−0.5 (2)	C11—N2—C12—S1	−177.78 (15)
C11—N3—N4—C13	171.33 (16)	N1—N2—C12—S1	−7.6 (3)
C11—C1—C2—C3	176.65 (15)	C17—N5—C13—N4	−57.1 (2)
C6—C1—C2—C3	−60.2 (2)	C14—N5—C13—N4	72.1 (2)
C7—C1—C2—C3	59.4 (2)	C12—N4—C13—N5	103.4 (2)
C1—C2—C3—C10	−59.7 (2)	N3—N4—C13—N5	−67.3 (2)
C1—C2—C3—C4	60.1 (2)	C13—N5—C14—C15	170.69 (16)
C10—C3—C4—C5	60.4 (2)	C17—N5—C14—C15	−58.3 (2)
C2—C3—C4—C5	−59.7 (2)	C18—N6—C15—C14	176.07 (17)
C3—C4—C5—C9	−59.7 (2)	C16—N6—C15—C14	−60.5 (2)
C3—C4—C5—C6	60.3 (2)	N5—C14—C15—N6	60.0 (2)
C9—C5—C6—C1	59.5 (2)	C18—N6—C16—C17	−179.65 (17)
C4—C5—C6—C1	−60.8 (2)	C15—N6—C16—C17	58.8 (2)
C11—C1—C6—C5	−179.12 (17)	C13—N5—C17—C16	−171.07 (15)
C2—C1—C6—C5	60.2 (2)	C14—N5—C17—C16	57.7 (2)
C7—C1—C6—C5	−58.0 (2)	N6—C16—C17—N5	−57.8 (2)
C11—C1—C7—C8	−178.13 (16)	C15—N6—C18—C19	−170.56 (18)
C6—C1—C7—C8	57.9 (2)	C16—N6—C18—C19	68.0 (2)
C2—C1—C7—C8	−60.5 (2)	N6—C18—C19—C24	28.6 (3)
C1—C7—C8—C10	61.0 (2)	N6—C18—C19—C20	−155.13 (18)
C1—C7—C8—C9	−58.6 (2)	C24—C19—C20—C21	0.4 (3)
C4—C5—C9—C8	58.9 (2)	C18—C19—C20—C21	−176.06 (19)
C6—C5—C9—C8	−61.4 (2)	C19—C20—C21—C22	−1.1 (3)
C7—C8—C9—C5	60.9 (2)	C20—C21—C22—C23	0.7 (3)
C10—C8—C9—C5	−58.4 (2)	C21—C22—C23—C24	0.3 (3)
C4—C3—C10—C8	−60.2 (2)	C20—C19—C24—C23	0.7 (3)
C2—C3—C10—C8	59.6 (2)	C18—C19—C24—C23	177.0 (2)
C7—C8—C10—C3	−60.3 (2)	C22—C23—C24—C19	−1.1 (3)
C9—C8—C10—C3	59.1 (2)	N2—N1—C25—C26	−178.69 (16)
N4—N3—C11—N2	1.70 (19)	N1—C25—C26—C27	178.57 (18)
N4—N3—C11—C1	−174.38 (16)	N1—C25—C26—C31	−0.6 (3)
C12—N2—C11—N3	−2.4 (2)	C31—C26—C27—O1'	−179.0 (3)
N1—N2—C11—N3	−173.33 (15)	C25—C26—C27—O1'	1.8 (4)
C12—N2—C11—C1	173.59 (17)	C31—C26—C27—C28	−1.6 (3)
N1—N2—C11—C1	2.6 (3)	C25—C26—C27—C28	179.19 (19)
C6—C1—C11—N3	−130.8 (2)	O1'—C27—C28—C29	179.2 (4)
C2—C1—C11—N3	−10.4 (2)	C26—C27—C28—C29	2.0 (3)
C7—C1—C11—N3	106.9 (2)	C27—C28—C29—C30	−0.3 (3)
C6—C1—C11—N2	53.8 (3)	C28—C29—C30—C31	−1.8 (3)
C2—C1—C11—N2	174.19 (17)	C29—C30—C31—O1	−177.4 (2)
C7—C1—C11—N2	−68.5 (2)	C29—C30—C31—C26	2.2 (3)
N3—N4—C12—N2	−0.9 (2)	C27—C26—C31—O1	179.11 (19)
C13—N4—C12—N2	−172.05 (16)	C25—C26—C31—O1	−1.7 (3)
N3—N4—C12—S1	178.77 (14)	C27—C26—C31—C30	−0.5 (3)
C13—N4—C12—S1	7.6 (3)	C25—C26—C31—C30	178.67 (19)

### Hydrogen-bond geometry (Å, º)

**Table:**

*D*—H···*A*	*D*—H	H···*A*	*D*···*A*	*D*—H···*A*
O1—H1*o*···N1	0.84	1.89	2.632 (2)	147
O1′—H1*o*′···N5^i^	0.84	1.92	2.714 (6)	158
C13—H13*A*···S1^ii^	0.99	2.68	3.650 (2)	166
C30—H30···N6^iii^	0.95	2.54	3.484 (3)	172

Symmetry codes: (i) −*x*+1/2, *y*−1/2, −*z*+3/2; (ii) −*x*+1/2, *y*+1/2, −*z*+3/2; (iii) −*x*+3/2, *y*−1/2, −*z*+3/2.
